# Separation of Volatile Fatty Acids by Simulated Moving Bed

**DOI:** 10.1021/acs.iecr.6c02420

**Published:** 2026-07-20

**Authors:** Cristiana Gomes, Ana M. Ribeiro, Alírio E. Rodrigues, Alexandre F. P. Ferreira

**Affiliations:** LSRE-LCM, ALiCE, Faculty of Engineering, University of Porto, Rua Dr. Roberto Frias, 4200-465 Porto, Portugal

## Abstract

This work evaluated the feasibility of the simulated moving bed (SMB) technology to fractionate volatile fatty acids produced through wastewater acidogenic fermentation. A lab-scale SMB unit equipped with 6 columns (FlexSMB), each packed with a nonfunctionalized resin (Amberlite XAD-4), was used to separate a binary solution of acetic and propanoic acids, for proof of concept. SMB operating conditions were first determined by building the separation region for a limit purity of 95% in both extract and raffinate streams, and a 1–2–2–1 column configuration. The process accomplished the separation of acetic and propanoic acids in the raffinate and extract streams, with purities of 97.8% and 100.0%, and recoveries of 100.0% and 97.8%, respectively. Productivities reached 2.93 and 2.81 mmol·g_ads_^–1^.day^–1^ in the raffinate and extract streams, respectively. An eluent consumption of 0.075 L·mmol^–1^ was attained. The mathematical model proposed accurately described the experimental data. Overall, SMB technology showed strong potential for efficient VFA fractionation.

## Graphical abstract

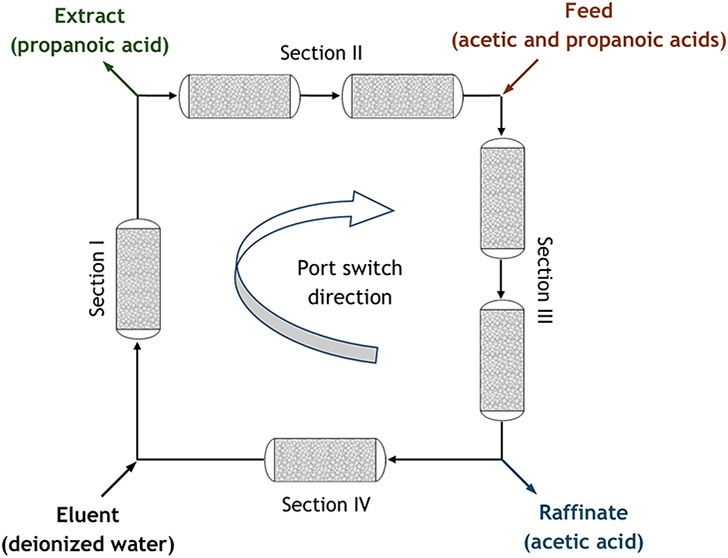

## 1 Introduction

Volatile fatty acids (VFA) are carboxylic acids with short chains of carbon, usually six or fewer carbon atoms. They are attractive and valuable components in the market since they can be used for the synthesis of alcohols, aldehydes, ketones, esters, olefins, etc., across several industries, such as food, pharmaceutical, chemical, cosmetics, bioenergy, and materials.^1–3^ The production of volatile fatty acids is usually achieved through chemical processes, specifically from petroleum-based feedstock, by the oxidation of the corresponding aldehyde. However, several problems are associated with these processes, such as negative health and environmental effects, the use of expensive catalysts, and extreme reaction conditions, which encourage the investigation of more sustainable processes.^1,4,5^ An alternative and widely studied approach is the production of volatile fatty acids via biological means, specifically via the acidogenic fermentation of organic matter present in various substrates, ranging from industrial or municipal wastewater to food or agricultural waste. This production method is a more cost-efficient process that can be carried out under more reasonable operating conditions than the chemical routes and also eliminates the accumulation of waste in the environment.^3,5^ However, not processed and mixed VFAs are less valuable than the refined and pure forms of individual acids. Therefore, it is required to separate them into a more worthy form to be used for their intended purpose.

An interesting and promising method for selectively separating the volatile fatty acids is adsorption. Over the years, some authors have investigated VFA fractionation in batch and fixed-bed systems. Fargues et al. associated two columns in series, each packed with a nonfunctionalized resin (Amberlite XAD-4) and a resin functionalized with a tertiary amine (Amberlyst A21) to remove carboxylic and aromatic solutes from industrial beet distillery effluents at pH 3–4. The Amberlite XAD-4 resin was able to completely retain the aromatic solutes and some part of the longer VFA, leaving the Amberlyst A21 resin to eliminate the remaining solutes (carboxylic solutes).^6^ In another study, the volatile fatty acids were selectively separated by a sequential adsorption–desorption process in batch mode with a nonfunctionalized resin (Dowex Optipore L-493) and a resin functionalized with a tertiary amine (Amberlite IRA-67). The nonfunctionalized resin was able to recover the longer acids (valeric and isovaleric acids), and then the weak ion-exchange resin retrieved the shorter acids (acetic and propanoic acids).^7^

Selective separation of specific components from a complex mixture is hard to accomplish with a single adsorption column or in batch adsorption reactors, especially if only low-selectivity adsorbents are available, high purities are required, and continuous operation is desired. True moving bed (TMB) is a continuous adsorption process able to achieve these types of separations. In a TMB process, the contact between the adsorbent and adsorbate is promoted by circulating both phases inside a column in a countercurrent mode, as illustrated in Figure 1. This process entails two inlet streams, the desorbent/eluent and the feed, and two outlet streams, the extract (with the more-adsorbed components) and the raffinate (where the less-adsorbed components are collected), which divide the unit into four sections. In section I (between the desorbent and extract nodes), the adsorbent is regenerated by the desorption of the more-adsorbed components. Sections II (between the extract and feed nodes) and III (between the feed and raffinate nodes) allow the component separation to happen. The less-adsorbed components are desorbed and transported with the liquid in the direction of the raffinate port to avoid contamination of the extract. Simultaneously, the adsorption of the more-adsorbed components takes place, which are being transported with the solid phase to prevent contamination of the raffinate. In section IV (between the raffinate and eluent nodes), the desorbent regeneration takes place through the adsorption of the less-adsorbed components, before being recycled to section I.^8,9^

**Figure 1:**
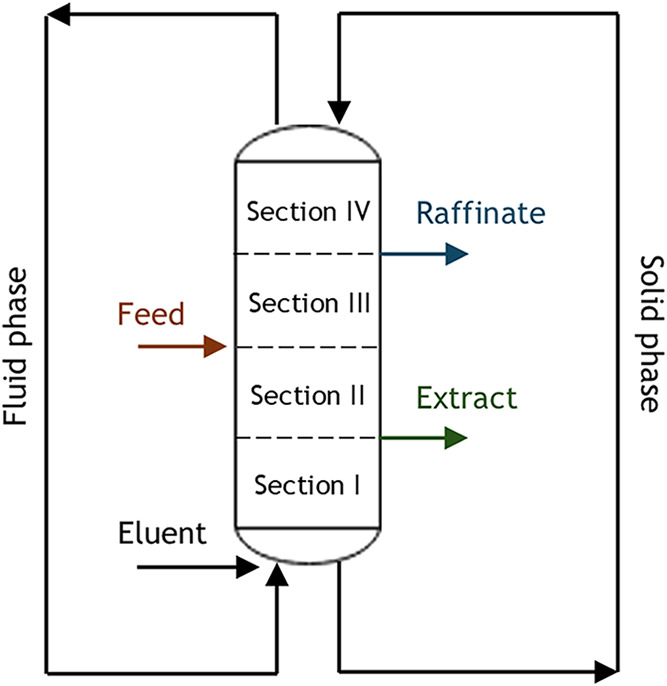
Schematic diagram of a true moving bed.

Due to technical difficulties in implementing a system with solid-phase movement, which leads to equipment abrasion, mechanical erosion of adsorbent, and difficulties in maintaining plug flow for the solid phase, simulated moving bed (SMB) technology was developed to overcome these limitations. SMB consists of a series of packed bed columns interconnected in a closed system, where the countercurrent contact between solid and liquid phases is simulated by periodically shifting, at regular time intervals (switching time, t*), the inlet and outlet ports in the direction of the liquid flow, as illustrated in Figure 2. When the inlet and outlet streams reach their initial positions, a complete cycle is achieved (one cycle is equal to *N*_c_ steps, where *N*_c_ is the total number of columns in an SMB unit).^8,9^

**Figure 2:**
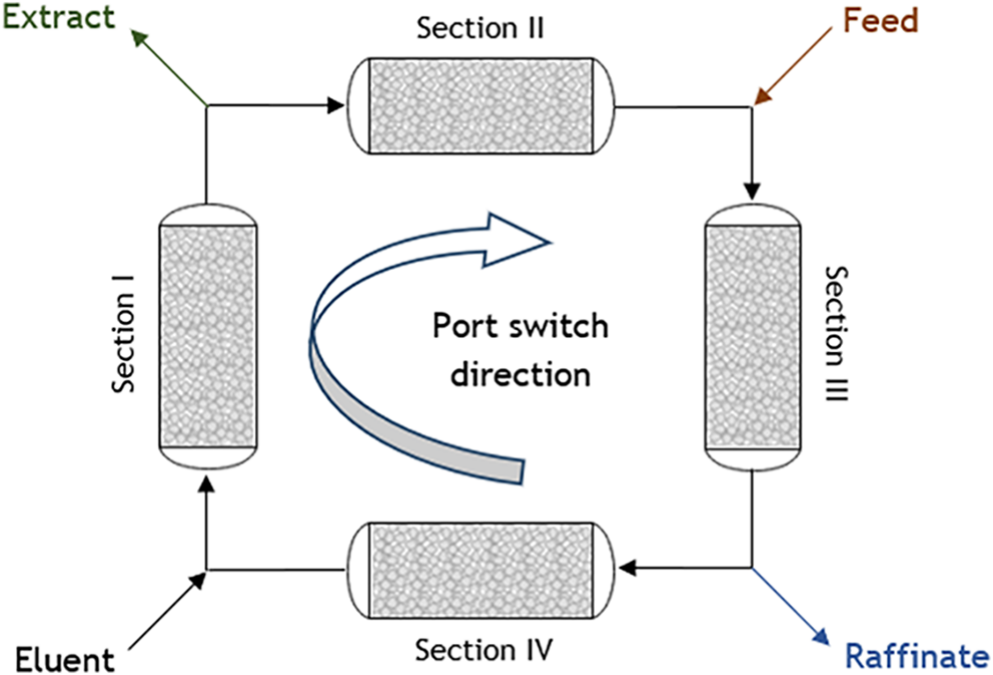
Schematic diagram of a simulated moving bed.

There are some studies in the literature on the design and optimization of simulated moving bed units for the recovery of carboxylic acids, mainly acetic and lactic acids, from model fermented solutions. Uribe Santos et al. designed an SMB unit (configuration 6–6–6–6) to separate acetic acid from a model fermented effluent, assuming salts and substrates are not adsorbed and using a weak-base ion-exchange adsorbent (Reillex 425). Acetic acid was recovered at high purity (>99%), high recovery (>95%), a productivity of 47.3 kg**·**m_ads_^–3^ **·**h^–1^ and 52.0 kg**·**m_ads_^–3^ **·**h^–1^, with desorbent consumption of 0.032 m^3^ **·**kg^–1^ and 0.020 m^3^ **·**kg^–1^, using methanol or water as desorbents, respectively.^10^ In another article by the same group, the recovery of acetic, lactic, and succinic acids from a model fermentation broth via the simulated moving bed process (configuration 6–6–6–6) was modeled, taking into account glucose adsorption and using the same adsorbent. The results showed that 95.8% of the acids can be recovered at 99.9% purity, with a productivity of 24.7 kg**·**m_ads_^–3^ **·**h^–1^ and an eluent consumption of 0.090 m^3^ **·**kg^–1^, using methanol as desorbent.^11^ Lee et al. experimentally explored the feasibility of an SMB unit, with a configuration 2–2–2–2 and packed with an anion-exchange resin (Reillex HP), for the separation of lactic and acetic acids in synthetic solutions at pH 2. Lactic and acetic acids were successfully fractionated at high purity and recovery (>95%).^12^

During acidogenic fermentation of wastewater, the volatile fatty acids usually produced in higher amounts are acetic and propanoic acids.^1,13^ In this context, the present work evaluates the separation of a binary mixture of acetic and propanoic acids using a simulated moving bed as a proof of concept for VFA fractionation. The objective is to assess the feasibility and separation efficiency of an SMB unit (FlexSMB) using Amberlite XAD-4, a nonfunctionalized adsorbent, while generating relevant knowledge to support the future treatment of more complex and real fermentation-derived VFA streams. The performance of this material was thoroughly investigated in batch and fixed-bed systems in a prior study, and the results can be consulted elsewhere.^14^ A mathematical model, previously validated for a fixed-bed system,^14^ was implemented to determine the separation region and select an operating point to perform the SMB experiment.

## 2 Experimental Methods

### 2.1 Chemicals and Materials

Acetic (Acet) and propanoic (Prop) acids were acquired from Sigma-Aldrich as standard reagent-grade products with purities of >99%. Synthetic solutions were prepared using deionized water. The material used was a commercial polystyrenedivinylbenzene nonfunctionalized ion-exchanger (Amberlite XAD-4), purchased from Sigma-Aldrich. The main physical properties of the adsorbent are summarized in Table 1. Before use, the resin was washed several times with deionized water to remove any impurities.

**Table 1:** Adsorbent’s Properties Provided by the Manufacturers

matrix structure	macroreticular
particle size, mm	0.490–0.690
pH range	0–14
maximum temperature, °C	150

### 2.2 Analytical Method

Samples collected were analyzed by high-performance liquid chromatography (HPLC) in a Shimadzu UFLC system (Japan) equipped with a Phenomenex RezexTM ROA-Organic Acid H+ (8%) (300 mm × 7.8 mm) column. The acid detection was accomplished by a DAD detector at 210 nm. A degassed and filtered mobile phase of 5 mM sulfuric acid was used in isocratic mode at 0.8 mL·min^–1^. The column temperature was kept at 60 °C, and the injection volume was 20 μL. Standard solutions of each acid at different known concentrations within the working concentration range, along with the equipment’s sensitivity signal, were analyzed in the HPLC to obtain the calibration curves.

### 2.3 SMB Setup and Operation

The Simulated Moving Bed unit used in this work was the installation presently available in the LSRE-LCM laboratory, labeled as FlexSMB and illustrated in Figure 3. This unit is equipped with six stainless steel columns (each one with 2 cm of internal diameter and 10 cm of length). Four HPLC pumps are used: one pump, positioned next to the raffinate node (in the beginning of section IV), allows the fluid to recycle into the system; another pump, placed in the extract line, collects the extract; and two other pumps are used to feed the system (one for the feed stream and another for the desorbent stream). The ceramic heads installed in the HPLC pumps allow a maximum flow rate of 50 mL**·**min^–1^ (eluent, extract and recycle pumps) and 10 mL**·**min^–1^ (feed pump). The SMB unit includes 6 SD (select-dead-end flow path) valves and 6 on–off valves (one per column), which direct the streams into the correct path, according to the configuration outlined. Relief valves were also installed after each pump to avoid overpressurization during the valves’ switching step. The automation and control routines of the SMB unit are ensured by using a LabVIEW (Laboratory Virtual Instrument Engineering Workbench) program. With this platform, the operator can program asynchronous port shifts to the inlets/outlets and adjust flow rates across the different sections.^15–17^ The FlexSMB was operated in a 1–2–2–1 configuration, where sections I and IV have one column each, and sections II and III have two columns each. Zones II and III are the separation zones and were assigned a higher number of columns to enhance the separation performance, whereas zones I and IV serve as regeneration zones for the adsorbent and the desorbent, respectively, and are well served by one column each. This configuration is a standard choice for six-column SMB systems.^18–21^

**Figure 3:**
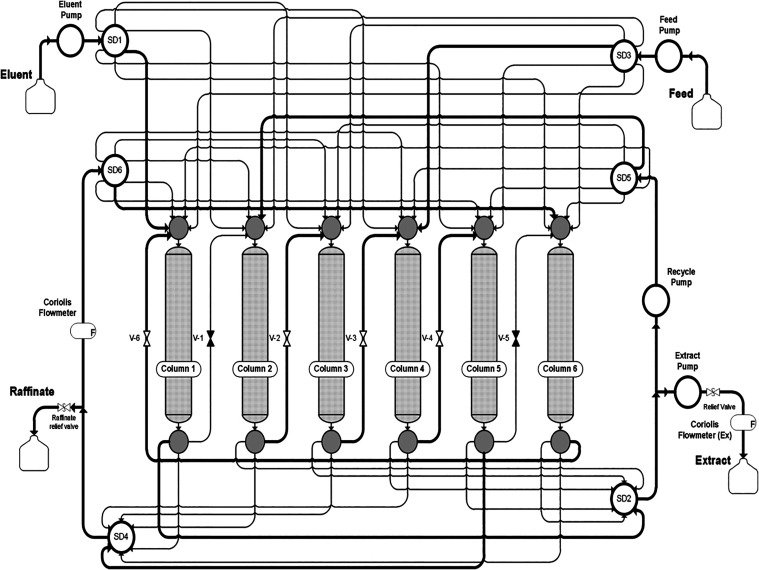
Scheme of the FlexSMB unit, during the first step of a 1–2–2–1 configuration. Reproduced with permission from ref 17. Copyright © 2012 WILEY-VCH Verlag GmbH & Co. KGaA, Weinheim.

The SMB columns were packed with the Amberlite XAD-4 resin and characterized by tracer experiments using Blue Dextran 2000 before installation in the FlexSMB unit. Due to its high molecular weight (≈2000 kDa) and high radius (around 30–40 nm), Blue Dextran 2000 does not penetrate into the porous structure of the adsorbent particles, allowing the determination of the bed porosity (interparticle bed void) and axial dispersion and the verification of uniform and reproducible packing across columns. An aqueous solution of Blue Dextran 2000 was continuously fed to the columns at 2.7 mL**·**min^–1^ in downward flow, at room temperature (20–25 °C). The outlet concentration was periodically monitored using a UV–vis spectrophotometer at 610 nm. Bed porosity (ε_b_), axial dispersion coefficient (*D*_ax_), residence time (τ), and Peclet number (*Pe*) were obtained by eqs 1 and 2 and by minimizing the absolute error of the cumulative distribution function (*F*(*t*)) calculated by eqs 3 and 4.^22,23^

1 $$\varepsilon_{b}=\frac{\tau }{V_{\mathrm{col}}/Q}$$

2 $$D_{\mathrm{ax}}=\frac{\mathrm{uL}}{\varepsilon_{b}\mathrm{Pe}}$$

3 $$F\left(t\right)=\frac{C_{t}}{C_{\mathrm{feed},t}}$$

4 $$F\left(t\right)=\int_{0}^{t}E\left(t\right)dt=\int_{0}^{t}\frac{\sqrt{\tau \mathrm{Pe}}}{\sqrt{4\pi t^{3}}}\exp\left(-\frac{\mathrm{Pe}{\left(\tau -t\right)}^{2}}{4\tau t}\right)dt$$

where *Q* is the total flow rate, *V*_col_ is the column volume, *u* is the superficial velocity, *L* is the column length, *C*_*t*_ is the outlet tracer concentration over time, *C*_feed,*t*_ is the tracer concentration in the feed stream, *E*(*t*) is the residence time distribution, and *t* is the time variable.

The SMB experiment was designed for the separation of a binary mixture of 80 mM acetic acid and 80 mM propanoic acid, at room temperature (20–25 °C). An equimolar mixture was chosen to provide a well-defined and reproducible reference system in which the adsorption behavior of the two acids on Amberlite XAD-4 can be well isolated and quantified. During the SMB operation, outlet flow rates were periodically measured to ensure stability and absence of fluctuations. Samples of the extract and raffinate streams were collected along each complete cycle and analyzed by HPLC. After achieving cyclic steady state, i.e., the acids’ outlet concentrations persisting constant over consecutive cycles, the concentration profiles were obtained by collecting samples at 25%, 50%, and 75% of the switching time.

## 3 SMB Modeling

### 3.1 Mathematical Model

The FlexSMB operation was designed according to the following mathematical model. The model assumes axial dispersion and plug flow for the fluid phase, isothermal operation, negligible pressure drop, constant porosity, and fluid velocity.^24^

Mass balance to the fluid phase in each column is given by

5 $$D_{\mathrm{ax}}\frac{\partial^{2}C_{i,k}}{\partial z^{2}}=\frac{u}{\varepsilon_{b}}\frac{\partial C_{i,k}}{\partial z}+\frac{\partial C_{i,k}}{\partial t}+\frac{\rho_{b}}{\varepsilon_{b}}\frac{\partial {\mathrm{q̅}}_{i,k}}{\partial t}$$

where *C*_*i*,*k*_ is the concentration of species *i* in the liquid phase in column *k*, *q̅*_*i*,*k*_ is the average adsorbed quantity of species *i* in column *k*, ρ_b_ is the bulk density, *z* is the axial distance variable from the column entrance, and *t* is the time variable.

The mass transfer inside the adsorbent particle is described by the linear driving force (LDF) model. This model is a simplified approach for describing intraparticle diffusion, assuming the adsorbent is a homogeneous particle and neglecting film mass transfer resistance.

6 $$\frac{d{\mathrm{q̅}}_{i,k}}{dt}=k_{\mathrm{LDF},i}\left(q_{i,k}^{*}-{\mathrm{q̅}}_{i,k}\right)$$

where *q*_*i*,k_^*^ is the adsorbed quantity of species *i* at equilibrium with *C*_*i*,*k*_ in column *k*, on dry mass basis, and *k*_LDF,*i*_ is the mass transfer coefficient of species *i*.

Adsorption equilibrium was described with the dual-site Langmuir model:

7 $$q_{i,k}^{*}=\frac{Q_{m1,i}K_{L1,i}C_{i,k}}{1+\sum_{j=1}^{n}K_{L1,j}C_{j,k}}+\frac{Q_{m2,i}K_{L2,i}C_{i,k}}{1+\sum_{j=1}^{n}K_{L2,j}C_{j,k}}$$

where *Q*_*m*1,*i*_ and *Q*_*m*2,*i*_ are the maximum adsorption capacities of species *i*, and *K*_*L*1,*i*_ and *K*_*L*2,*i*_ are the Langmuir constants of species *i*, for each active site in the dual-site Langmuir model.

Mass balances were also written for all nodes (between columns):^24^

Eluent node:

8 $$Q_{I}=Q_{D}+Q_{\mathrm{rec}}=Q_{D}+Q_{\mathrm{IV}}$$

9 $$Q_{\mathrm{IV}}C_{i,\mathrm{IV},\mathrm{out}}=Q_{I}C_{i,I,\mathrm{in}}$$

Extract node:

10 $$Q_{\mathrm{II}}=Q_{I}-Q_{X}$$

11 $$C_{i,I,\mathrm{out}}=C_{i,\mathrm{II},\mathrm{in}}$$

Feed node:

12 $$Q_{\mathrm{III}}=Q_{\mathrm{II}}+Q_{F}$$

13 $$Q_{\mathrm{II}}C_{i,\mathrm{II},\mathrm{out}}=Q_{\mathrm{III}}C_{i,\mathrm{III},\mathrm{in}}-Q_{F}C_{i,F}$$

Raffinate node:

14 $$Q_{\mathrm{IV}}=Q_{\mathrm{III}}-Q_{R}$$

15 $$C_{i,\mathrm{III},\mathrm{out}}=C_{i,\mathrm{IV},\mathrm{in}}$$

Nodes between other columns:

16 $$C_{i,k,\mathrm{out}}=C_{i,k+1,\mathrm{in}}$$

Global mass balance:

17 $$Q_{D}+Q_{F}=Q_{X}+Q_{R}$$

where *Q*_I_, *Q*_II_, *Q*_III_, and *Q*_IV_ are the flow rates in sections I, II, III, and IV, respectively; *Q*_D_, *Q*_X_, *Q*_F_, *Q*_R_, and *Q*_rec_ are the liquid phase flow rates of desorbent, extract, feed, raffinate, and recycling streams, respectively; *C*_I,i,in_, *C*_II,i,in_, *C*_III,i,in_, and *C*_IV,i,in_ are the inlet concentrations of species *i* in sections I, II, III, and IV, respectively; *C*_I,i,out_, *C*_II,i,out_, *C*_III,i,out_, and *C*_IV,i,out_ are the outlet concentrations of species *i* in sections I, II, III, and IV, respectively; and *L*_*k*_ is the length of column *k*.

The initial and boundary conditions are^24^

Initial conditions:

18 $$t=0\to C_{i,k}={\mathrm{q̅}}_{i,k}=0$$

Boundary conditions:

19 $$z=0\to uC_{i,k,\mathrm{in}}=uC_{i,k}-\varepsilon_{b}D_{\mathrm{ax}}\frac{\partial C_{i,k}}{\partial z}$$

20 $$z=L_{k}\to \frac{\partial C_{i,k}}{\partial z}=0$$

In addition to the mass balances, the mathematical model also takes into consideration the dead volumes in the system to enhance the accuracy of describing the experimental SMB operation. Pipping was represented as transfer lines, assuming plug flow, and the port switching delays, which represent 0.5% of the switching time, were also accounted.^15,24^

The numerical solution of the SMB mathematical model was attained using gPROMS model builder version 7.0.7 software. The second-order orthogonal collocation method (OCFEM) was applied to discretize the axial dimension of the bed columns in 40 finite elements, and the system of differential equations was solved by using the DAEBDF integrated solver. A tolerance of 10^–5^ was set. It was assumed that the SMB simulation reached the cyclic steady state when the mass balance for each component closed within 5% between two consecutive cycles. Adsorption equilibrium and kinetics data were determined in a prior study and can be consulted elsewhere.^14^

### 3.2 Separation Region

The design of an SMB unit involves the determination of several parameters, such as flow rates, switching time, and column configuration. Equilibrium theory can be used as a basis to determine the feasible operating region that allows a complete separation and to select suitable operating conditions. This theory defines a set of constraints that delimit the separation region based on the operation of the SMB process and the function of each section, considering the adsorption equilibrium effects and neglecting mass transfer resistance. These constraints are written in terms of fluid and solid flow rates, using the dimensionless parameter γ_*j*_ (eq 21), and are defined by eqs 23–26.^25,26^

21 $$\gamma_{j}=\frac{\left(1-\varepsilon_{b}\right)}{\varepsilon_{b}}\frac{Q_{j}}{Q_{s}}$$

where *Q*_*j*_ is the flow rate of the fluid phase in section *j* and *Q*_s_ is the solid flow rate, which is defined according to eq 22.

22 $$Q_{s}=\frac{\left(1-\varepsilon_{b}\right)V_{\mathrm{col}}}{t^{*}}$$

Section I: more retained species (Prop) need to move with the liquid for solid regeneration.

23 $$\mathrm{section}\;I:,\gamma_{1}>\frac{{\mathrm{q̅}}_{\mathrm{Prop},I}\rho_{b}}{C_{\mathrm{Prop},I}}\frac{1}{\varepsilon_{b}}$$

Sections II and III: more retained species (Prop) need to go with the solid phase (go to the extract), and the less retained species (Acet) with the liquid phase (go to the raffinate).

24 $$\mathrm{section}\;\mathrm{II}:,\gamma_{2}>\frac{{\mathrm{q̅}}_{\mathrm{Acet},\mathrm{II}}\rho_{b}}{C_{\mathrm{Acet},\mathrm{II}}}\frac{\left(1-\varepsilon_{b}\right)}{\varepsilon_{b}}\wedge \gamma_{2}<\frac{{\mathrm{q̅}}_{\mathrm{Prop},\mathrm{II}}\rho_{b}}{C_{\mathrm{Prop},\mathrm{II}}}\frac{\left(1-\varepsilon_{b}\right)}{\varepsilon_{b}}$$

25 $$\mathrm{section}\;\mathrm{III}:,\gamma_{3}>\frac{{\mathrm{q̅}}_{\mathrm{Acet},\mathrm{III}}\rho_{b}}{C_{\mathrm{Acet},\mathrm{III}}}\frac{\left(1-\varepsilon_{b}\right)}{\varepsilon_{b}}\wedge \gamma_{3}<\frac{{\mathrm{q̅}}_{\mathrm{Prop},\mathrm{III}}\rho_{b}}{C_{\mathrm{Prop},\mathrm{III}}}\frac{\left(1-\varepsilon_{b}\right)}{\varepsilon_{b}}$$

Section IV: less retained species (Acet) moves with the solid phase to clean the eluent that will be recycled to section I.

26 $$\begin{array}{ll} \mathrm{section}\;\mathrm{IV}:,\gamma_{4}<\frac{{\mathrm{q̅}}_{\mathrm{Acet},\mathrm{IV}}\rho_{b}}{C_{\mathrm{Acet},\mathrm{IV}}} & \frac{1}{\varepsilon_{b}} \end{array}$$

where *C*_Acet,*j*_ and *C*_Prop,*j*_ are the concentrations in the fluid phase of acetic and propanoic acids, respectively, in section *j*, and *q̅*_Acet,*j*_ and *q̅*_Prop,*j*_ are the average adsorbed quantities of acetic and propanoic acids, respectively, in section *j*.

Equilibrium theory is commonly used to obtain an initial estimate of the separation region, which can subsequently be refined using more detailed models. This region is defined as the area in a γ_2_ × γ_3_ plot where both extract and raffinate are pure, i.e., the plot delimited by constraints of eqs 24 and 25, maintaining the switching time and flow rates in sections I and IV fixed. Figure 4 shows a typical separation region in the plane γ_2_ × γ_3_ that can be found for an SMB operation. Besides the region where both extract and raffinate are pure, three other regions are defined: two where only one of the streams (raffinate or extract) is pure, and another where neither of the extract nor raffinate streams is pure. The SMB unit must be operated at operating conditions within the region where both raffinate and extract are pure, especially near the vertex point, which represents the best operating conditions in terms of productivity and eluent consumption.^25,26^

**Figure 4:**
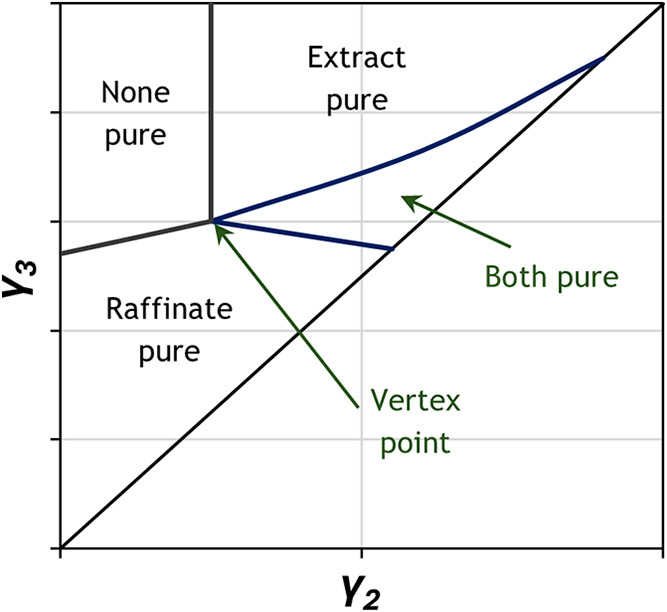
Scheme of a typical separation region for an SMB unit.

An algorithm was implemented in the gPROMS software to build the separation region based on the mathematical model presented in Section 3.1 First, initial estimates of the flow rates of sections I and IV were obtained from the equilibrium theory (eqs 23 and 26), for an arbitrary switching time. These flow rates were then adjusted to avoid any contamination of the adsorbent and eluent phases, while the switching time was modified to reduce the flow rates and ensure compliance with the operating limits of the pumps. The separation region, represented by the γ_2_ × γ_3_ plot, was determined by systematically varying the feed flow rate and identifying the corresponding extract and raffinate flow rates that satisfy the predefined product purity specifications. The values that did not fulfill the requirements were discarded.^26,27^

### 3.3 Performance Parameters

The SMB performance and process feasibility were assessed over complete cycles according to the following equations:^24^

Purity (solvent-free):

27 $${\mathrm{Pur}}_{X}=\frac{{\mathrm{C̅}}_{\mathrm{Prop},X}}{{\mathrm{C̅}}_{\mathrm{Acet},X}+{\mathrm{C̅}}_{\mathrm{Prop},X}},\begin{array}{l} {\mathrm{Pur}}_{R}=\frac{{\mathrm{C̅}}_{\mathrm{Acet},R}}{{\mathrm{C̅}}_{\mathrm{Acet},R}+{\mathrm{C̅}}_{\mathrm{Prop},R}} \end{array}$$

Recovery:

28 $${\mathrm{Rec}}_{X}=\frac{Q_{X}{\mathrm{C̅}}_{\mathrm{Prop},X}}{Q_{F}{\mathrm{C̅}}_{\mathrm{Prop},F}},{\mathrm{Rec}}_{R}=\frac{Q_{R}{\mathrm{C̅}}_{\mathrm{Acet},R}}{Q_{F}{\mathrm{C̅}}_{\mathrm{Acet},F}}$$

Productivity (in mmol·g_ads_^–1^·day^–1^):

29 $${\mathrm{Prod}}_{X}=\frac{Q_{X}\left({\mathrm{C̅}}_{Ac\mathrm{et},X}+{\mathrm{C̅}}_{\mathrm{Prop},X}\right)}{N_{c}V_{\mathrm{col}}\rho_{b}},{\mathrm{Prod}}_{R}=\frac{Q_{R}\left({\mathrm{C̅}}_{Ac\mathrm{et},R}+{\mathrm{C̅}}_{\mathrm{Prop},R}\right)}{N_{c}V_{\mathrm{col}}\rho_{b}}$$

Eluent consumption (in L·mmol^–1^):

30 $$\mathrm{DC}=\frac{Q_{D}}{Q_{F}\left({\mathrm{C̅}}_{\mathrm{Acet},F}+{\mathrm{C̅}}_{\mathrm{Prop},F}\right)}$$

where *C̅*_i,X_, *C̅*_i,R_, and *C̅*_i,F_ are the average concentrations of species *i* in the extract (X), raffinate (R), and feed (F) streams over a cycle, respectively, and subscript *i* refers to the more retained (propanoic acid) and less retained (acetic acid) components present in the feed, extract, and raffinate streams. The average concentration of species *i* in a given stream (*C*) is determined by *C̅*_*i*,C_ = ∫_*t*_^*t* + *N*_c_ t*^ *C*_i,C_ *dt*/*N*_c_ t*.

## 4 Results and Discussion

### 4.1 Fractionation of Acetic and Propanoic Acids

The six columns of the FlexSMB unit were packed with the Amberlite XAD-4 adsorbent and characterized through a tracer experiment with Blue Dextran 2000. Figure 5 shows the experimental results for all columns, as well as the fitting of eq 4 (dashed line). The bed parameters obtained are summarized in Table 2. Bed density was determined based on the known column volume and the adsorbent mass packed.

**Figure 5:**
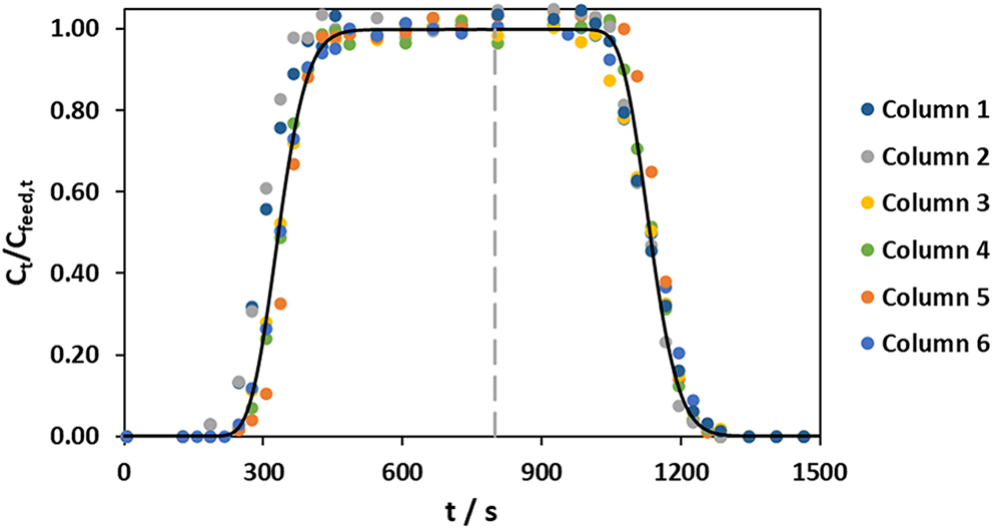
Blue Dextran 2000 breakthrough curves in each FlexSMB column. The points represent experimental data, and the full line is the respective model simulation obtained with the average parameters’ values.

**Table 2:** Characteristics of Each FlexSMB Column

column length, *L* (dm)	1.0
column inner diameter, *d*_int_ (dm)	0.2
column volume, *V*_col_ (dm^3^)	3.14 × 10^–2^
bed density, ρ_b_ (g·dm^–3^)	400
Peclet number, *Pe*	93
residence time, τ (s)	336.0
bed porosity, ε_b_	0.48
dispersion coefficient, *D*_ax_ (dm^2^·s^–1^)	3.21 × 10^–5^

The separation region was determined for a minimum purity requirement of 95% for both the extract and the raffinate. The switching time was set to 30 min. The eluent and recycle flow rates were set to 22.81 and 1.50 mL·min^–1^, respectively, avoiding contamination of sections I and IV. By running the algorithm described in Section 3.2, the separation region represented in Figure 6 was obtained. An operation point was selected to perform the SMB assay (red point in Figure 6), and the operating conditions are summarized in Table 3.

**Figure 6:**
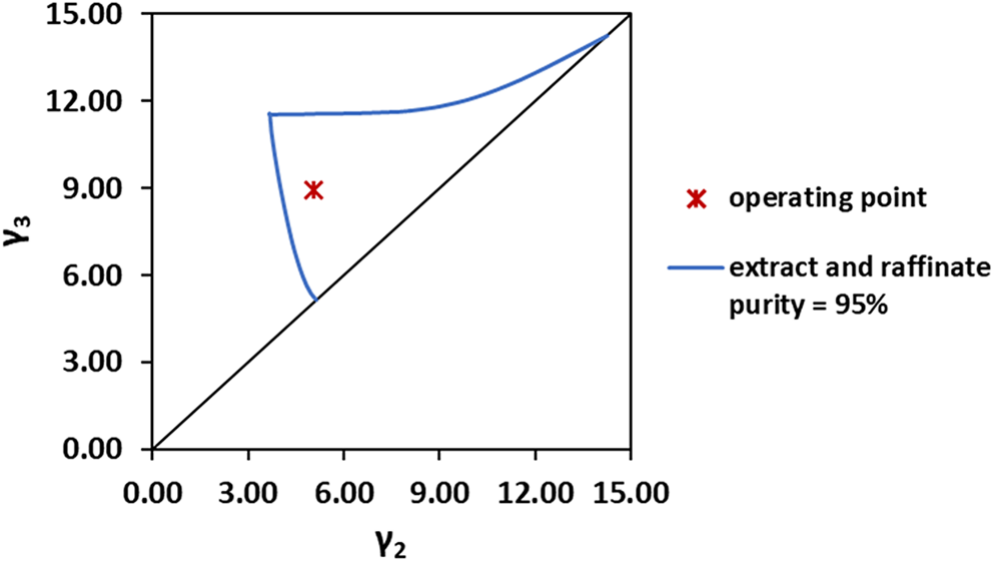
Simulated separation region for 95% purity of the extract and raffinate streams, for the separation of acetic and propanoic acids in a binary solution, as well as the selected operating point (red point).

**Table 3:** Operating Conditions Used in the SMB Assay

time switch, t* (min)	30
feed flow rate, *Q*_F_ (mL·min^–1^)	1.95
eluent flow rate, *Q*_D_ (mL·min^–1^)	22.81
extract flow rate, *Q*_X_ (mL·min^–1^)	21.76
raffinate flow rate, *Q*_R_ (mL·min^–1^)	3.00
recycle flow rate, *Q*_rec_ (mL·min^–1^)	1.50
acetic acid feed concentration, *C*_*F*,Acet_ (mM)	80
propanoic acid feed concentration, *C*_*F*,Prop_ (mM)	80
configuration	1–2–2–1

The histories of the average concentrations in the extract and raffinate streams obtained during the SMB assay are presented in Figure 7. The dashed lines in the figure are simulation results.

**Figure 7:**
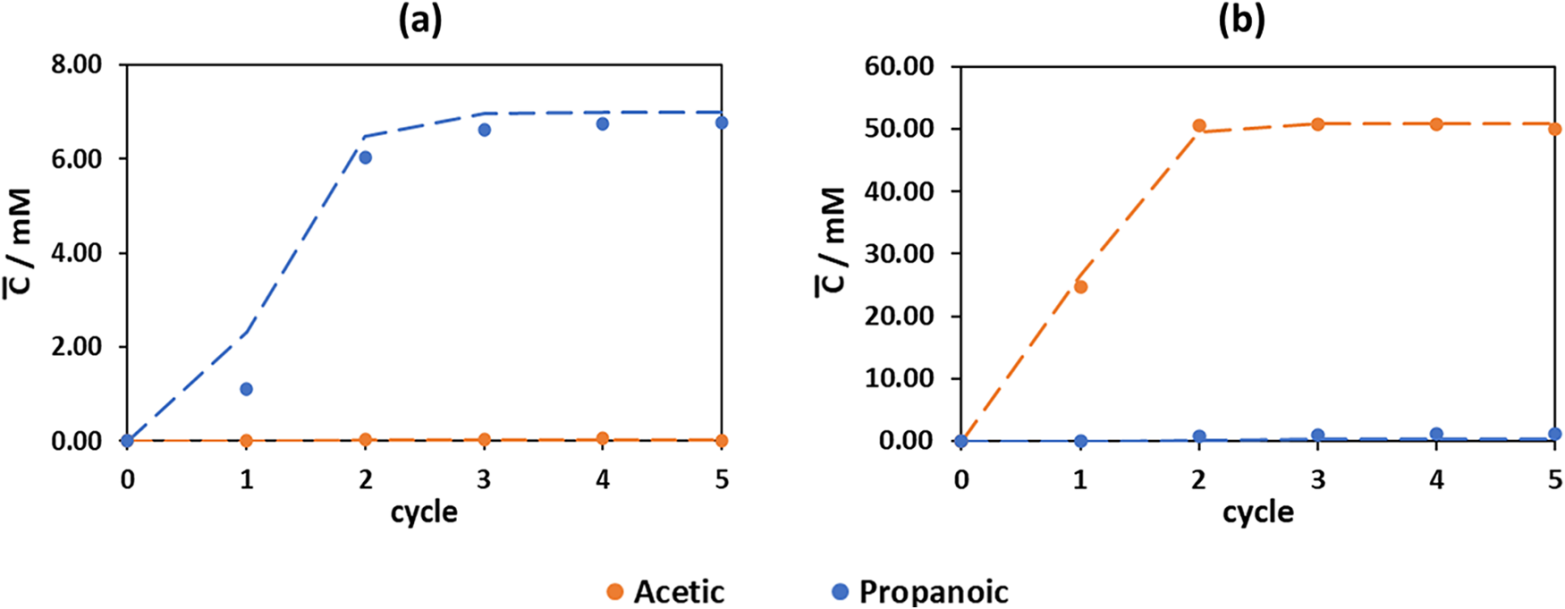
History of the outlet average concentrations of extract (a) and raffinate (b) streams along each cycle. The points represent experimental data, and the dashed line is the respective model simulation.

A cyclic steady state was achieved after 5 cycles of operation since the acids’ outlet concentrations remained constant over the last cycles. At this point, the concentration profiles were determined at 25%, 50%, and 75% of the switching time and are illustrated in Figure 8, along with the mathematical model prediction (full lines).

**Figure 8:**
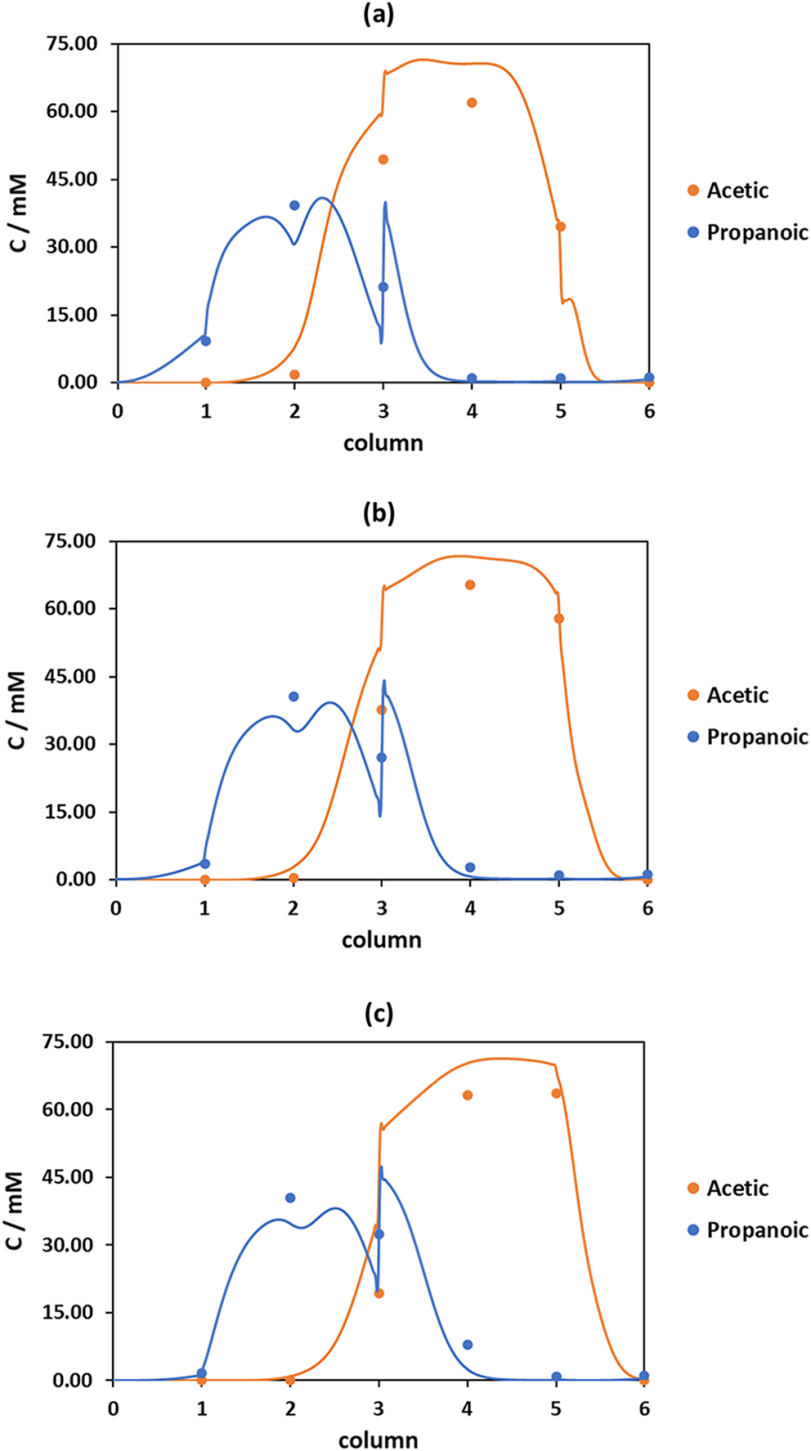
Internal concentration profiles at (a) 25%, (b) 50%, and (c) 75% of the switching time, at cyclic steady state. The points represent experimental data, and the full line is the respective model simulation.

The concentration profiles obtained indicate that propanoic acid is retained by the solid and collected in the extract stream, whereas acetic acid moves preferentially with the liquid phase and is recovered in the raffinate stream, as expected. Additionally, the mathematical model’s predictions were in good agreement with the experimental results.

In this experiment, the acetic and propanoic acids were separated at 97.8% and 100.0% purity and 100.0% and 97.8% recovery in the raffinate and extract, respectively. The productivity values were 2.93 mmol·g_ads_^–1^·day^–1^ in the raffinate stream and 2.81 mmol·g_ads_^–1^·day^–1^ in the extract stream. An eluent consumption of 0.075 L·mmol^–1^ was attained. These performance parameters’ values are summarized in Table 4, along with the simulation results, which are very similar to each other.

**Table 4:** SMB Performance Parameters

	experimental		simulation	
	extract	raffinate	extract	raffinate
purity (%)	100.0	97.8	99.8	99.6
recovery (%)	97.8	100.0	99.6	99.8
productivity (mmol·g_ads_^–1^·day^–1^)	2.81	2.93	2.90	2.93
eluent consumption (L·mmol^–1^)	0.075		0.075	

### 4.2 Process Considerations

The FlexSMB experiment performed in this work allowed the validation of the mathematical model to describe the VFA adsorption/desorption in conventional simulated moving bed systems and the proof of concept for the separation of volatile fatty acids with this technology. Although the binary mixture investigated in this study represents a simplified system, it provides relevant evidence of the potential of SMB for the selective fractionation of VFA and establishes a basis for future studies with more complex streams.

Conventional SMB systems are mainly used for binary separations, as only two outlet streams are produced: the extract (with the more-adsorbed components) and the raffinate (with the less-adsorbed components). Thus, to achieve a multicomponent separation with this technology, alternative and nonconventional SMB operating modes are used, such as SMB in cascade. In a simulated moving bed cascade process, multiple SMB units are connected in series to separate the components of a mixture sequentially. In each unit, one target component is purified, while the remaining mixture is passed to the next SMB stage for further separation. Various design strategies are possible, ranging from isolating a single component per SMB unit to separating the mixture into binary fractions, which are further processed in a standard simulated moving bed mode.^28,29^

The recovery and selective separation of acetic and propanoic acids from fermented effluents can be achieved by employing an SMB in a cascade system. Two configurations are possible: (i) a first SMB unit recovers the acetic and propanoic acids from a mixture of the seven volatile fatty acids typically produced (acetic, propanoic, butyric, isobutyric, valeric, isovaleric, and hexanoic acids), followed by a second unit that fractionates the resulting binary mixture into individual acetic acid and propanoic acid streams, and (ii) a first SMB unit removes the acetic acid from the mixture, and then the propanoic acid recovery is accomplished by a second unit. A thorough study of the design of a cascaded simulated moving bed for complete VFA separation is still required and will be the subject of future work.

## 5 Conclusions

The separation of acetic and propanoic acids in an equimolar binary solution (80 mM each) was conducted in an SMB unit with 6 columns (FlexSMB), using a 1–2–2–1 configuration and a nonfunctionalized resin (Amberlite XAD-4) as the solid phase and water as the desorbent. The operating conditions were determined by building the separation region with a minimum purity of 95% for both extract and raffinate and a switching time of 30 min. The FlexSMB was operated under the flow rates of 22.81, 1.50, 1.95, 3.00, and 21.76 mL·min^–1^ for the eluent, recycle, feed, raffinate, and extract streams, respectively. The cyclic steady state was achieved after 5 cycles of operation. Acetic and propanoic acids were separated and collected in the raffinate and extract streams, with purities of 97.8% and 100.0%, respectively, and recoveries of 100.0% and 97.8%, respectively. The productivity obtained was 2.93 mmol·g_ads_^–1^·day^–1^ in the raffinate stream and 2.81 mmol·g_ads_^–1^·day^–1^ in the extract stream. An eluent consumption of 0.075 L·mmol^–1^ was attained. The performance parameters, concentration profiles, and histories acquired experimentally were in good agreement with the mathematical model simulations, allowing the model to be validated for a simulated moving bed system.

Overall, these results confirmed the feasibility of volatile fatty acid separation using simulated moving bed technology and highlighted its potential for the fractionation of VFA produced through acidogenic fermentation.

## Nomenclature

**List of Symbols**
*C*: concentration in the liquid phase, mol·m^–3^
*C*_feed_: concentration in the feed stream, mol·m^–3^
*C*_in_: inlet concentration, mol·m^–3^
*C*_out_: outlet concentration, mol·m^–3^
*C̅*: average concentration, mol·m^–3^
*D*_ax_: axial dispersion coefficient, m^2^·s^–1^
*DC*: eluent consumption, m^3^·mol^–1^
*d*_int_: column inner diameter, m
*E*(*t*): residence time distribution, –
*F*(*t*): cumulative distribution function, –
*K*_L1_: Langmuir constant for active site 1 in the dual-site Langmuir model, m^3^·mol^–1^
*K*_L2_: Langmuir constant for active site 2 in the dual-site Langmuir model, m^3^·mol^–1^
*k*_LDF_: LDF coefficient or mass transfer coefficient, s^–1^
*L*: column length, m
*N*_c_: total number of columns in an SMB unit, –
*Pe*: Peclet number, –
Prod: productivity, mol·kg_ads_^–1^·s^–1^
Pur: purity, –
*Q*: flow rate of the liquid phase, m^3^·s^–1^
*Q*_s_: flow rate of the solid phase, m^3^·s^–1^
*Q*_m1_: maximum adsorption capacity for active site 1 in the dual-site Langmuir model, mol·kg_ads_^–1^
*Q*_m2_: maximum adsorption capacity for active site 2 in the dual-site Langmuir model, mol·kg_ads_^–1^
*q**: adsorbed quantity at equilibrium, mol·kg_ads_^–1^
*q̅*: average adsorbed quantity in the adsorbent, mol·kg_ads_^–1^
Rec: recovery, –
*t*: time variable, s
*t**: switching time, s
*u*: superficial velocity, m·s^–1^
*V*_col_: column volume, m^3^
*z*: axial distance variable from the column entrance, m
**Greek Letters**
γ: ratio of the interstitial flow rates, –
ε_b_: bed porosity, –
ρ_b_: bed density, kg·m^–3^
τ: residence time, s
**Subscripts and Superscripts**
C: stream (X—extract, R—raffinate, F—feed, D—eluent, rec—recycle)
i: species
j: section (I, II, III, IV)
k: column
t: tracer
